# Dendritic cells during Epstein Barr virus infection

**DOI:** 10.3389/fmicb.2014.00308

**Published:** 2014-06-20

**Authors:** Münz Christian

**Affiliations:** Viral Immunobiology, Institute of Experimental Immunology, University of ZurichZurich, Switzerland

**Keywords:** plasmacytoid dendritic cells, conventional dendritic cells, monocyte-derived dendritic cells, natural killer cells, T cells

## Abstract

Epstein Barr virus (EBV) causes persistent infection in more than 90% of the human adult population and is associated with 2% of all tumors in humans. This γ-herpes virus infects primarily human B and epithelial cells, but it has been reported to be sensed by dendritic cells (DCs) during primary infection. These activated DCs are thought to contribute to innate restriction of EBV infection and initiate EBV-specific adaptive immune responses via cross-priming. The respective evidence and their potential importance for EBV-specific vaccine development will be discussed in this review.

## INFECTION AND TUMORIGENESIS BY EPSTEIN BARR VIRUS

Epstein Barr virus (EBV) was discovered 50 years ago in a cell line (EB1) from an African child with Burkitt’s lymphoma (Epstein et al., 1964). Despite this association with lymphomas and carcinomas, including Hodgkin’s lymphoma and nasopharyngeal carcinoma (Kutok and Wang, 2006; Cesarman, 2014), EBV is carried without symptoms by the vast majority of persistently infected individuals, which account for more than 90% of the adult human population (Rickinson et al., 2014). EBV-associated malignancies arise with increased frequency in immunosuppressed patients, for example after transplantation (post-transplant lymhoproliferative disease or PTLD), immunosuppressive co-infections such as HIV, or primary genetic immunodeficiencies (like X-linked lymphoproliferative disease or XLP). These findings indicate that asymptomatic chronic infection with EBV results in part from continuous virus-specific immune control. Mainly cellular immunity by natural killer (NK) and T cells seems to mediate this immune control (Rickinson et al., 2014), and some EBV-associated malignancies can even be cured by adoptive transfer of EBV-specific T-cell lines (Gottschalk et al., 2005). Some evidence has been provided that dendritic cells (DCs) sense EBV infection and are involved in the priming of these protective innate and adaptive immune responses. This evidence and its relevance for EBV-specific vaccine development will be discussed in this review.

## SELECTIVE HOST CELL TROPISM OF EBV

Dendritic cells are probably not initiating EBV-specific immune control after getting directly infected by the virus. Although it has been reported that EBV can enter monocyte precursors of DCs, no EBV antigen expression could be found in these studies and only CMV-promoter-driven green fluorescent protein (GFP) expression of recombinant EBV was detected after infection (Li et al., 2002; Guerreiro-Cacais et al., 2004). Indeed, the main host cell of EBV is the human B cell. In healthy EBV carriers, memory B cells seem to constitute the site of long-term persistence (Babcock et al., 1998). Latency 0 in these memory B cells is associated with no viral protein expression but transcription of EBV encoded small RNAs (EBERs) and micro RNAs (miRNAs). EBV uses its envelope glycoprotein gp 350 to attach to complement receptors 1 and 2 (CD35 and CD21) on the surface of B cells, uses gp42 binding to MHC class II molecules and finally the trimeric complex of gH, gL, and gB for fusion with the membrane (Connolly et al., 2011). The B-cell compartment is reached by EBV after transmission via saliva in the tonsils. Naïve B-cell infection at these sites is associated with the expression of eight latent EBV proteins and the non-translated RNAs (Babcock et al., 2000). This latency III or growth program drives infected B cells into proliferation and is present in PTLD and HIV-associated diffuse large B cell lymphomas (DLBCL). The six EBV nuclear antigen (EBNA1, 2, 3A, 3B, 3C, and LP) and two latent membrane proteins (LMP1 and LMP2) are sufficiently immunogenic, so that tumors expressing all of these only emerge under severe immunosuppression. One outcome of this EBV-driven activation of naïve B cells is thought to be their differentiation into germinal center B cells. In these centroblasts and centrocytes, only three EBV proteins retain their expression (EBNA1, LMP1, and LMP2; Babcock et al., 2000). This latency II pattern, which is also found in Hodgkin’s lymphoma, was proposed to rescue EBV-infected B cells from deletion by mimicking B-cell receptor engagement and T-cell help via CD40 signaling by LMP2 and LMP1, respectively. Therefore, EBV rescues its infected B cells from the germinal center reaction in order to gain access into the long-lived memory B-cell pool. From there, the virus reactivates into lytic replication and infectious particle production after B-cell receptor stimulation. Indeed, lytic EBV replication has primarily been found in plasma cells (Laichalk and Thorley-Lawson, 2005) and B-cell receptor cross-linking can initiate replication in some Burkitt’s lymphoma cell lines (Takada, 1984). If this reactivation occurs in mucosal secondary lymphoid tissues, the virus can be secreted into saliva and transmitted to new individuals.

Efficient transmission, however, might require an additional amplification step in epithelial cells, which have been found to be preferentially infected by free virus from the basolateral side (Tugizov et al., 2003). Integrin binding by BMRF2 and gH/gL for gB-mediated fusion might mediate this epithelial cell infection and B-cell-produced EBV seems to be particularly good at it (Borza and Hutt-Fletcher, 2002). This basolateral infection during shedding into saliva might give rise to the EBV-associated carcinomas at mucosal sites, including nasopharyngeal carcinoma. The biased tropism of EBV toward B and epithelial cells suggests that DCs are most likely not directly infected by EBV, but process viral particles and dying EBV-infected B and epithelial cells for both immune detection of infection and initiation of innate and adaptive immune responses.

## INNATE IMMUNE RECOGNITION OF EBV

**FIGURE 1:**
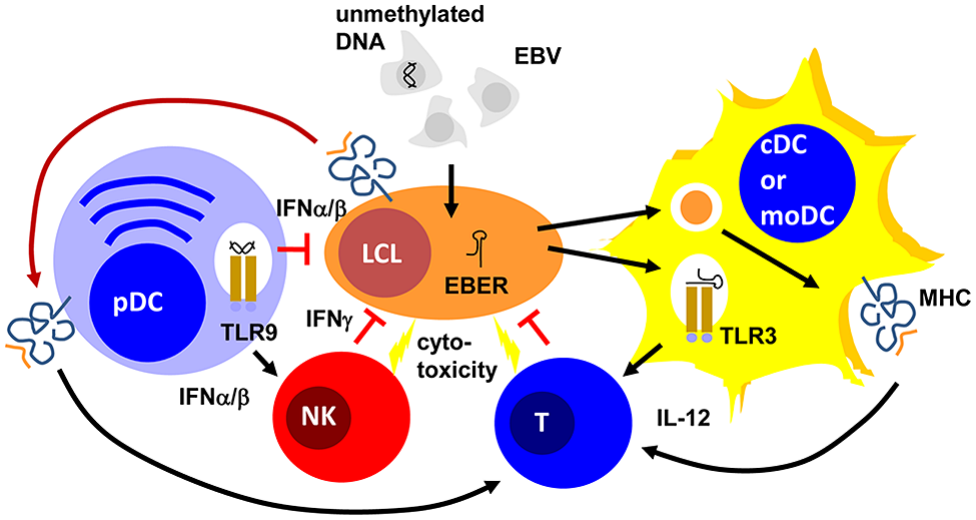
**Plasmacytoid, conventional and monocyte-derived DCs might contribute to EBV specific immune control.** Unmethylated DNA of EBV particles and EBERs of EBV-infected B cells (LCLs) mature plasmacytoid (pDCs) and conventional or monocyte-derived DCs (cDCs or moDCs) via TLR9 or TLR3 stimulation, respectively. These mature pDC and cDC or moDC populations activate natural killer (NK) and T cells via type I interferon (IFNα/β) or interleukin 12 (IL-12) secretion, respectively. For T-cell stimulation by MHC presentation they acquire EBV antigens either via phagocytosis of dying LCLs (for cDCs and moDCs) or trogocytosis of EBV epitope presenting MHC complexes (pDCs). The activated NK and primed T cells then delay primary EBV infection via IFNγ and kill infected cells. PDCs can also delay primary EBV infection via IFNα/β production.

Epstein Barr virus is a double-stranded DNA virus of the lymphocryptovirus subgroup of γ-herpesviridae. As such, the viral particle carries double-stranded linear DNA without much methylation, which can be detected by the toll-like receptor (TLR) 9 (Casanova et al., 2011). Indeed, EBV DNA triggers TLR9-mediated recognition of the virus in plasmacytoid DCs, B cells, and monocytes (Fiola et al., 2010; Severa et al., 2013; Younesi et al., 2014). Once EBV enters B cells, it circularizes its DNA to episomes, which then get heavily methylated (Woellmer et al., 2012). Therefore, viral DNA of dying EBV-infected B cells is probably invisible to TLR9. In contrast to mice, human conventional DCs (cDCs) do not express TLR9 (Iwasaki and Medzhitov, 2004). Instead TLR2 and 3 have been implicated in EBV recognition by macrophages and conventional DCs (Gaudreault et al., 2007; Ariza et al., 2009; Iwakiri et al., 2009). While the TLR2 ligand of EBV remains enigmatic, EBERs have been proposed as TLR3 ligands (Iwakiri et al., 2009). It appears that EBERs are released from infected B cells in complex with the EBER-binding protein La. Apart from TLR3-binding, EBERs can also stimulate the intracellular pathogen associated molecular pattern (PAMP) receptor retinoic acid-inducible gene 1 (RIG-I; Samanta et al., 2006). Both TLR-3 and RIG-I recognize double-stranded RNA (dsRNA) and EBERs seem to form hairpin structures that allow their recognition by these two intra- and extracellular receptors for dsRNA. Therefore, EBV seems to stimulate both pDCs and cDCs by viral DNA in viral particles and viral RNA released from infected cells, respectively (**Figure 1**).

## INNATE IMMUNE CONTROL OF EBV

These DC populations seem to play significant roles during primary EBV infection. Along these lines pDCs are potent sources of type I interferons (IFNα and β; Reizis et al., 2011). In particular, human pDCs produce high levels of IFNα2 and α14 (Meixlsperger et al., 2013). IFNα and β have been found to restrict B-cell transformation by EBV during the first 24 h of infection (Lotz et al., 1985). While this study suggested that the protective type I IFN effect directly targeted infected B cells, a PBMC transfer model into SCID mice suggested that the IFNα/β-dependent effect was mediated via NK cell activation and EBV-specific memory T cells (Lim et al., 2006). In this study, PBMC reconstituted SCID mice were challenged with EBV infection with and without prior deletion or enrichment of pDCs in the transferred PBMCs. They observed pDC- and TLR9-dependent IFNα production in response to primary EBV infection. Furthermore, EBV-induced lymphoma formation was observed after pDC depletion and this was mediated by decreased NK and EBV-specific memory T-cell activation in the transferred PBMCs of healthy EBV carriers. Therefore, type I IFN, probably produced primarily by pDCs during primary EBV infection, seems to have a protective function against EBV-induced B-cell transformation, early by directly targeting B cells and later by activating protective lymphocyte populations.

One of these protective lymphocyte populations are NK cells. Their activity is stimulated by DCs during viral infections in mice (Lucas et al., 2007). In particular, surface presentation of IL-15 is important for this NK cell activation by DCs. Similarly, human DCs are able to activated NK cells (Ferlazzo et al., 2002). IL-12, IL-15, and IFNα are primarily involved in NK cell activation by human monocyte-derived DCs (moDCs; Ferlazzo et al., 2004; Strowig et al., 2008). This NK cell activation occurs most potently after TLR3-mediated maturation of moDCs and preferentially stimulates CD56^bright^ killer immunoglobulin-like receptor (KIR)-negative NK cells (Brilot et al., 2007; Strowig et al., 2008). In tonsils, the primary site of EBV infection, this NK cell subset produces large amounts of type II IFN (IFN; Strowig et al., 2008; Lünemann et al., 2013). IFNγ can restrict primary B-cell transformation by EBV during the first 3–4 days (Lotz et al., 1985; Strowig et al., 2008; Lünemann et al., 2013). It seems to delay LMP1 expression during the first 3–5 days after primary EBV infection of B cells (Strowig et al., 2008). Accordingly, DC stimulation of NK cells restricts B-cell transformation by EBV *in vitro*, especially when the NK cells are derived from tonsils and are part of the CD56^bright^KIR^-^ NK cell subset (Strowig et al., 2008; Lünemann et al., 2013). Apart from this cytokine-mediated delay of B-cell transformation, NK cells might also directly kill infected B cells undergoing lytic EBV replication (Pappworth et al., 2007; Chijioke et al., 2013). This restricts lytically EBV replicating B cells *in vitro* and *in vivo* in a mouse model of human immune component reconstitution after CD34^+^ hematopoietic progenitor cell (HPC) transfer (Pappworth et al., 2007; Chijioke et al., 2013). In this mouse model, NK cell activation can be also achieved by TLR3 agonist injection (Strowig et al., 2010) and this adjuvant elicits potent DC maturation (Meixlsperger et al., 2013). Thus, DCs mediate innate immune control during EBV infection by IFNα/β production of pDCs and activate NK cells that delay B-cell transformation via IFNγ and eliminate lytic EBV replication by killing of virus-producing cells (**Figure 1**).

## DCs IN THE PRIMING OF ADAPTIVE EBV-SPECIFIC IMMUNE CONTROL

Apart from innate lymphocyte activation during EBV infection, DCs are most likely also involved in the priming of EBV-specific, protective T-cell responses (Rickinson et al., 2014). Indeed, *in vitro* EBV infection of B cells is very inefficient in priming EBV-specific T cells from PBMCs of EBV-negative donors (Bickham et al., 2003). However, addition of autologous moDCs allows priming of EBV-specific T cells in these cultures. For this purpose, DCs presumably cross-present EBV antigens from dying EBV-infected B cells in these cultures. Indeed, such dying EBV-transformed B cells can be presented on MHC class I and II molecules of moDCs for CD8^+^ and CD4^+^ T-cell stimulation, respectively (Münz et al., 2000; Subklewe et al., 2001). However, some observations call this prominent role of DCs in the priming of EBV-specific T-cell responses into question. For example, EBV-transformed lymphoblastoid B cell lines (LCLs) were able to prime EBV-specific CD4^+^ T cells at low frequencies, but these could be expanded after CD25 targeted selection (Savoldo et al., 2002). Furthermore, it was found that CD8^+^ T cells primarily recognize early, but not late lytic EBV antigens, apart from some prominent latent EBV antigens (Hislop et al., 2007). Indeed, only subdominant CD8^+^ T-cell responses were documented against late lytic EBV antigens (Abbott et al., 2013), while CD4^+^ T-cell responses against late lytic antigens can be observed (Adhikary et al., 2006). Since EBV encoded inhibitors of MHC class I antigen presentation get expressed during early viral gene expression and, therefore, would primarily prevent late lytic antigen presentation on MHC class I, this hierarchy in lytic EBV antigen recognition by CD8^+^ T cells was taken as an indication that EBV infected cells prime this CD8^+^ T-cell hierarchy. An alternative explanation, however, could be that DCs prime these different EBV specificities similarly by cross-presentation, and the preference for early lytic EBV antigen recognition then is established by amplification of the respective T-cell responses via restimulation by EBV-infected B cells. A similar amplification was recently observed for the EBNA1 antigen targeted to the endocytic receptor DEC-205 on DCs and B cells (Leung et al., 2013b). Among the human DC subsets, priming of EBV-specific T-cell responses has been ascribed or demonstrated primarily for phagocytic DC subsets. These would include CD1c^+^ or CD141^+^ cDCs, and moDCs. However, a recent study also reported that pDCs might trogocytose MHC class I peptide complexes, presenting EBV epitopes (Bonaccorsi et al., 2014). This cross-dressing with LCL-derived MHC class I complexes is also sufficient to stimulate EBV-specific CD8^+^ T cells. Therefore, different DC populations could contribute to EBV-specific T-cell priming to establish protective EBV-specific immune control in healthy carriers of this human tumor virus.

## CONCLUSION AND OUTLOOK

These EBV-specific T cells are clearly the protective entity during the adaptive immune responses against EBV (Rickinson et al., 2014). How they are primed requires further investigation, because vaccination against EBV should probably engage the respective DC populations both by adjuvant choice as well as antigen targeting to the relevant DC subsets. Indeed with the advent of mice with reconstituted human immune system compartments, which recapitulate primary EBV infection and EBV-associated lymphomagenesis (Leung et al., 2013a), it becomes feasible to define DC populations that are involved in the priming of protective immune responses *in vivo*. In this preclinical model, CD4^+^ and CD8^+^ T cells mediate immune control over EBV infection and B-cell lymphoma development (Strowig et al., 2009) and protective EBV-specific CD4^+^ T cells can be primed with vaccine candidates (Gurer et al., 2008; Meixlsperger et al., 2013). Therefore, it should be feasible to define important DC populations that initiate EBV-specific immune control by for example antibody depletion (Meixlsperger et al., 2013), in order to then refine vaccination approaches that protect from EBV infection challenge. With such smart vaccine formulations that are directed against the most relevant DC populations EBV negative adolescents with a high risk to suffer symptomatic EBV infection could be vaccinated and their predisposition to develop Hodgkin’s lymphoma or multiple sclerosis attenuated (Hjalgrim et al., 2003; Thacker et al., 2006).

## Conflict of Interest Statement

The author declares that the research was conducted in the absence of any commercial or financial relationships that could be construed as a potential conflict of interest.
